# Physiopathology of vesico-ureteral reflux

**DOI:** 10.1186/s13052-016-0316-x

**Published:** 2016-11-29

**Authors:** Salvatore Arena, Roberta Iacona, Pietro Impellizzeri, Tiziana Russo, Lucia Marseglia, Eloisa Gitto, Carmelo Romeo

**Affiliations:** 1 Department of Human Pathology in Adult and Developmental Age “Gaetano Barresi” - Unit of Paediatric Surgery, University of Messina, 98125 Messina, Italy; 2 Unit of pediatric Surgery, Oxford University Hospital, Oxford, UK; 3 Department of Human Pathology in Adult and Developmental Age “Gaetano Barresi” - Unit of Paediatrics, University of Messina, Messina, 98125 Italy

**Keywords:** Vesicoureteral reflux, Active antireflux mechanism, Passive antireflux mechansim, Interstitial cells of Cajal, Sarcoglycan

## Abstract

Vescico-Ureteral Reflux (VUR) is a common condition in childhood, caused by a congenital anomaly at the Vescico-Ureteral Junction (VUJ) level. It seems that the main cause could be an abnormal embryological development occurred during the early stage of fetal life.

Refluxing ureteral endings show structural and functional anomalies: previous studies have shown a significant decrease in alfa actin, miosin and desmin contents as well as an high rate of atrophy and muscular degeneration with disorganized muscular fibres. The roles played by Cajal cells and Connexin 43 in generating peristaltic waves appears to be fundamental for the physiological VUJ function and activity. Attention was focused also on the congenital muscular deficiency of the RUs, on regard to general morphology, smooth muscle cells architecture, inflammatory markers and the distribution of collagen composition.

This review will discuss and investigate the importance of the modified configuration of Sarcoglycan (SG) sub complex (particularly the deficiency of the ε-SG and the increased expression of the α-SG), the role played by Cajal Cells, the intravescical tunnel length to ureteral diameter ratio as possible causes of the functional alterations in the refluxing ureteral ends leading towards the VUJ incompetence.

## Background

Primary Vescico-Ureteral Reflux (VUR) is a common condition likely related to a congenital anomaly of the Vescico-Ureteral Junction (VUJ) caused by its abnormal embryological development [1]. VUJ represents the border-line through the upper urinary tract, characterized by low pressure level, and the lower urinary tract, characterized instead by high pressure [2]. It acts therefore, in protecting the upper tract from reflux using both active and passive anti-reflux mechanisms [2].

The exact incidence of the VUR in the neonatal population still remains unknown because of the invasive radiology required for early diagnosis.

Overall, it is reported to be as low as 1 to 2% but it might be higher [3].

Data about the incidence of VUR are reported in the current literature as 25 to 40% of children presenting with urinary tract infection and in 3 to 19% of infants with hydronephrosis diagnosed on antenatal ultrasound scan screening [3].

### Passive anti-reflux mechanism

The most common explanation for a competent anti-reflux mechanism is represented by a passive compression of the ceiling of the intravesical ureter against the underlying detrusor, According to this theory, the intravesical length to ureter and its diameter is considered to be fundamental in maintaining the VUJ closure and preventing VUR.

Specifically, the relationship between the length of the intravesical ureter and its diameter is reported to be the tipping point supporting the ‘passive’ reflux defence mechanism [4].

For this reason, many authors considered the laterality of the intravesical ostium and the shortness of the ureteric transmural and submucosal course relative to its diameter as the main cause of VUR [5]. As a consequence, the spontaneous resolution of VUR would be caused by the bladder growth, thanks to the submucosal tunnel elongation [5]. However, Oswald et al. [6, 7] reported an intravesical length of newborn ureters of 3017.2 ± 388.9 μm and a diameter of 1354.5 ± 231.3 μm, which implies that the ratio of the intravesical ureteric length to intravesical ureteric diameter is 2.23 : 1.

Moreover, in fetuses between 11 and 20 weeks of gestation the intravesical ureteric length to diameter ratio decrease to 0.69:1 and 1.23:1, respectively [7]. This evidence could explain the higher incidence and severity of VUR in neonates and premature and, also, the tendency of VUR to solve spontaneously.

### Active antireflux mechanism

Because of a lower intravesical ureteric length to diameter ratio than expected, it was suggested that an intrinsic event might play a role to impair the so-called “active antireflux mechanism”.

Active shortening of the longitudinal muscle layer of the transmural and submucosal ureter areas drive off the urine bolus into the bladder. Functional and structural alterations of ureteric ends seem to impair the active valve mechanism of the VUJ, causing VUR [8, 9].

The smooth muscle cells, therefore act by transforming the extra-cellular matrix (ECM) through the extracellular production of proteinase and their inhibitors [6]. As a consequence, smooth muscular cells could have a deep impact also in the progression of the maturation for the refluxing ureters. Evaluating the turn-over of the ECM, and specifically of collagen I and III, it was reported the role played by the Metalloproinase of the matrix (MMPs). The MMPs are secreted by connective cells of mesenchymal origin like fibroblasts, myoblasts and macrophage CD 68 positive [6]. The abnormal function of the smooth muscle cells and the alteration of the micro-environment of the ECM have driven towards the immunochemical evalutation of S-100 protein as an innervation marker of the ureteral wall.

A study conducted on 36 distal refluxing ureteral ends (RUs), showed an increased expression of MMP1 in CD68 cells in the smooth muscle cells and in the fibroblasts as well as a significant decrease of the neural cells S-100 in the positive RUs [6].

The defective innervation of the distal ureteral endings therefore, could be considered as fundamental for the modification of the active anti-reflux mechanism.

The regular peristaltic movement is thus essential to drive the urine bolus from the kidney to the bladder towards the normal peristaltic ureters.

Many authors have reported as dysplasia, atrophy and architectural derangement of smooth muscle cells in RUs is essential for the deficient active valve mechanism [2]. Tokunaka et al. first introduced the term of muscular dysplasia to describe the anomalies of the intravescical distal ureteral tract in VUR patients [10].

Reduced smooth muscle fascicles and its defective configuration, creates an uncoordinated muscular contraction accompanied by the loss of c-kit-positive interstitial cells of Cajal (ICCs) at the VUJ level [6, 11, 12]. Such cells were identified by immunostaining for the c-kit protooncogene at the cell membrane. The ICCs are cells considered as responsible for pacemaker activity in the human ureter, and coordinating ureteric motility [13–15]. These cells produce electrical slow-wave potential and propagate peristaltic activity. A decrease in ICC density, is closely associated with a various motility disorders [16, 17], as well known in patients with colonic slow-transit constipation [18]. Many Authors [11, 12] reported the presence of a few ICCs in patients with VUR.

Their main function is to generate automatic rhythm for coordinate peristalsis, allowing also anterograde movement of urine bolus towards the ureters [15, 19]. An inverse correlation has been reported between the severity of VUR and the loss of ICCs in refluxing ureteric ends [11, 12]. It is not clear why the depletion of c-kit-expressing ICCs occurs in refluxing ureteric ends. Interestingly, mammalian ICCs derive from smooth muscle progenitors, whose differentiation is independent from neural crest-derived cell lines [20].

In refluxing ureteric ends it has been detected a grade-correlated defect of muscle cells, among which c-kit-positive ICCs differentiate inside the ureteric ending, so that the loss of c-kit-positive ICCs might be a consequence of the disruption of muscle cells. Conversely, as mesenchymal cells, sorround the mesonephric duct, differentiate into the ureteric inner layer of smooth muscle cells [21], the delayed elongation of the Wolffian duct endings might be a pathogenetic event for muscular and subsequent ICC defects in VUR. Thus, the delayed maturation of ureteric ends is coherent with a possible spontaneous resolution of VUR, after postnatal remodelling of the VUJ, as shown for the pathogenesis of primary megaureter [22].

It has been suggested that the loss of c-kit-positive ICCs could be also secondary to ureteric trauma during episodes of VUR, as reported in the proximal segment of obstructed fetal bowel [23]. Consistently it is acknowledged that the mechanical stress can affect the expression of developmental genes, providing evidence that molecular signals are not the only forces involved in modelling the developing embryo [24]. Moreover, the reduction of Connexin 43 has been reported. It is considered as the most important hexamerous that constitute the gap junction, essential for the intracellular signal. The lacking of Connexin 43 might lead to a disfunction of the smooth muscle cells activity, explaining the ureteral dismotility and the VUR [11].

As regards, manometry on refluxing ureteric showed a significant decrease in both P max and basal pressure in refluxing ureteral endings, and a highly significant negative correlation of these values with the grade of VUR.

Moreover in high-grade VUR, RUs showed a severe impairment of basal pressure and ureteral arrhythmia and in the worst scenario, a ‘silent’ pressure profile pattern. In fact, in high grade VUR (IV to V) the manometry showed a severe impairment of basal pressure and ureteral arrhythmia in 73% of RUs, and a “silent” pressure profile pattern in 27% [12].

The variable and inconsistent pressure of the peristaltic waves, and its irregular wave rhythm, are likely to result in disturbed urine transport along the distal part of RUs. It has been suggested that the silent ureter might represent an advanced stage of ureteric arrhythmia, suggesting a more damaged ureter that resembles a ureteric arrhythmic state [11].

The histological and histochemical findings of the RUs showed a muscular disarrangement and atrophy, and increased interstitial fibrosis, significantly correlated with the grade of VUR.

Gearhart et al. [25] reported a degree of smooth muscle deterioration and more collagen deposition in dilated ureters with primary VUR. The ureteric refluxing ends showed a proportion of muscle and collagen decreased from normal (1 :0.3) to 1:3 [7]. Furthermore, Oswald et al. [6] reported a replacement of muscle bundles by connective tissue, leading to ureteric strictness. It is apparent that any defect of the longitudinal muscle coat implies an impairment of the active valve mechanism, with subsequent VUR.

Indeed, the impossibility of sufficient contraction of the ureteric muscular layer produces VUR, by preventing the closure of the ureteric orifice [5]. The significant impairment of ureteric pressure is obvious, and positively correlates with the VUR grade. The reduction of basal pressure is associated with muscle disarrangement or atrophy and subsequent interstitial fibrosis in refluxing ends, leading to loss of muscular mass and ureteric stiffening. Depending on the low density of c-kit-positive ICCs, shortened irregular (bicuspid) and intermittent peristaltic waves were recorded, with quiescent periods, severe impairment of peristalsis, until there was a silent manometric pattern [12]. It has also been supposed that an impairment of overall microperfusion in Refluxing Ureter ends, would lead to tissue ischemia, and diminished ureteral perfusion are likely to induce and support smooth muscle cells dysfunction and apoptosis, so as a result, that functional and structural alterations may further deteriorate the active valve mechanism of the ureterovesical junction VUJ, causing VUR [5].

Micro vessel density (MVD) is markedly reduced in the RUs, because of a possible alteration of the blood stream that is accompanied by a decrease level of Vascular Endothelial Growth Factor (VEGF), that could represent the primary cause. The loss of VEGF could lead to the alteration of the smooth muscle cells and could play an important role in the reflux mechanisms [8].

Immunohistochemical data and RT PCR study demonstrated a significant VUR grade relation with the deficiency of ε-SG and with the increased expression of α-SG [26]. It was supposed that the different behaviour of SG subunits could be explained in at least 2 ways.

The first hypothesis is that the SG subcomplex could play a key role in the physiopathology of VUR: the deficiency of any of the SGs would lead to a specific loss of the SG subcomplex, destabilizing the alfa destroglycan, and causing the loss of the membrane integrity and fiber degeneration [27].

Furthermore, this subcomplex is critical for conferring stability to the muscle membrane, thus, protecting the sarcolemma from stress that develops during muscle fiber contraction [28].

The latter hypothesis instead suggest that the structural deficiency of the trigonal VUJ, could cause a passive stretching of refluxing urine on the ureter with derangement of the SG subcomplex. This effect can also be explained by reduced amplitude of contraction and relaxation of the smooth muscle fibers of the ureter caused by structural deficiency and altered ureteral peristalsis. This condition could lead to a loss of mechanical stress transmitted over cell surface receptors that physically couple the cytoskeleton to the ECM or to other cells. Therefore, mechanical signals could be integrated with other environmental signals and transduced into a biochemical response through force dependent changes in scaffold geometry or molecular mechanisms by multimodular tensegrity architecture [26, 28]. Resultant changes in the topology of these networks could alter cellular biochemistry directly. Consequently, these mechanical changes could cause modifications of chemical signals, although variations of the structural pathway of the SG subcomplex and α-SG could replace or compensate the loss of ε-SG.

In fact, an altered configuration of the SG complex, in particular the deficiency of ε-SG and over expression of α-SG, could explain the structural and functional changes in RUs, displaying smooth muscle cell apoptosis, smooth muscular atrophy, increased interstitial fibrosis, hypoperistaltic or ureteroarrhythmic manometric pattern and, at least, the impaired overall microperfusion, diminishing the active valve mechanism of the VUJ.

The altered expression of SGs, causing a structural instability to the muscular plasmatic membrane might promote stress damage that develops during smooth muscle fiber contraction in RUs, loss of membrane integrity, smooth muscle cell apoptosis and fiber degeneration.

Moreover, the loss of ε-SG could explain the manometric findings in the VUJ of RUs, including hypoperistalsis and ureteroarrhythmia or “silent” pattern [26].

It is noteworthy that the depletion of c-kit positive ICCs in RUs might be the consequence of the disruption of smooth muscle cells, progenitors of ICCs, or secondary to mechanical stress, affecting the expression of developmental genes, during episodes of VUR [12, 26].

Moreover, loss of ε-SG in RUs might explain the overall microperfusion, ongoing functional and structural alterations of vesicoureteral junction, and subsequent deterioration of the active antireflux mechanism [26].

## Conclusion

The pathogenesis of primary VUR still remains unclear.

It seems that a possible mechanism on its development could be research during the early embryonic phase.

The most important anomaly seems to occurred at the VUJ level.

Several studies have shown how the reduction of smooth muscle fascicles and the defective configuration at VUJ level, creates an uncoordinated muscular contraction also accompanied by the loss of c-kit-positive ICCs.

The role of ICCs, infact should be related to the maintainance of normal peristaltic waves driving the urine bolus towards an anterograde way.

Focusing on the smooth muscle cells of the VUJ, the action of some drugs as alpha-blockers or anticholinergics could results in beneficial effects in the treatment of VUR associated to dysfunctional voiding [29, 30].

Changes at ECM have been widely discussed and reported in the current literature, and it seems that an important role is played by the modification of sarcoglicans especially in the development of distal tract of refluxing ureters.

Further studies are needed in order to have a better understanding of the antireflux mechanisms.

## Abbreviations

ECM: Extracellular matrix
ICCs: Interstitial cells of Cajal
SG: Sarcoglycan
VUJ: Vesicoureteral juntion
VUR: Vesicoureteral reflux
